# Inferior Vena Cava Tumor Thrombus Secondary to Mucinous Adenocarcinoma of the Stomach

**DOI:** 10.7759/cureus.16543

**Published:** 2021-07-21

**Authors:** Nicholas Tuck, Syed Kamran

**Affiliations:** 1 Internal Medicine, University of Kansas School of Medicine, Wichita, USA

**Keywords:** inferior vena cava tumor thrombus, mucinous tumours, gastric malignancy, invasive mucinous adenocarcinoma, venous thrombus

## Abstract

The expansion of a tumor into a blood vessel is known as a tumor thrombus. Tumor thrombi are caused by many types of cancers and commonly confer a poor prognosis. Tumor thrombus secondary to non-hepatic gastrointestinal cancers are rare and when reported is typically seen in the portal vein rather than the inferior vena cava (IVC). Presentation specifically of gastric malignancies is usually non-specific and late in the disease course, thus limiting treatment options. In this case we present a patient with an inferior vena cava tumor thrombus secondary to mucinous adenocarcinoma of the stomach.

## Introduction

The expansion of a tumor into a blood vessel is known as a tumor thrombus. Tumor thrombi are caused by many types of cancers including hepatocellular carcinoma (HCC), Wilms tumors, adrenal cortical carcinoma, and renal cell carcinoma, but are rarely seen in primary gastric carcinoma. Only 1.2% of patients with gastric cancer were found to have a portal vein thrombus on autopsy [1]. When discovered, tumor thrombus remarkably alters the stage, prognosis and overall outcomes [2]. For example, in patients with early, localized gastric cancer who undergo surgical resection and chemotherapy, five-year survival can be as high as 90% [3], whereas portal vein thrombosis related to gastric cancer has a median survival of approximately five months [1]. Symptom presentation of a tumor thrombus is largely variable and depends on the primary malignancy but can include extremity swelling, cardiac dysfunction, Budd-Chiari syndrome and can even be asymptomatic. This is evident as patients typically present late in the disease course, with up to 90% having invasive or metastatic disease on presentation [1]. In this case we present a patient with an inferior vena cava (IVC) tumor thrombus secondary to mucinous adenocarcinoma of the stomach.

## Case presentation

The patient was a 74-year-old male with past medical history of hypertension, diabetes mellitus, hyperlipidemia, and chronic obstructive pulmonary disease on chronic oxygen therapy who presented with dizziness. Symptoms started one week prior to admission causing frequent lightheadedness when standing resulting in one atraumatic fall. He denied syncope, weakness, or neurologic dysfunction. Other pertinent history included a 13-pound weight loss over the prior one to two months and a 40-pack year smoking history. He reported adherence with all his medications and no recent medication changes.

Physical exam showed diminished bilateral breath sounds on respiratory auscultation but was otherwise unremarkable. Initial vitals showed a blood pressure of 96/64 mmHg and an oxygen saturation of 94% on 3L of supplemental oxygen. Pertinent labs showed a serum creatine of 1.74 mg/dL and mild elevation of aspartate and alanine transaminase (45 and 56 U/L respectively); the remaining laboratory findings were unremarkable.

**Figure 1:**
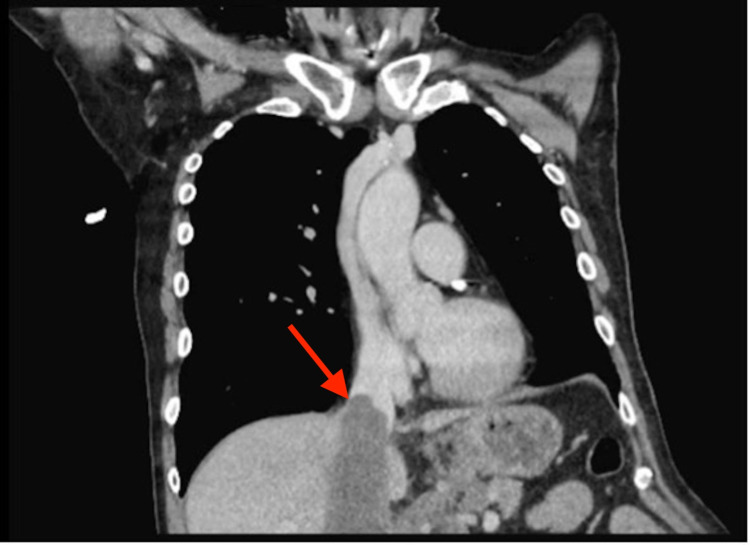
CT scan with frontal view of inferior vena cava thrombus

**Figure 2:**
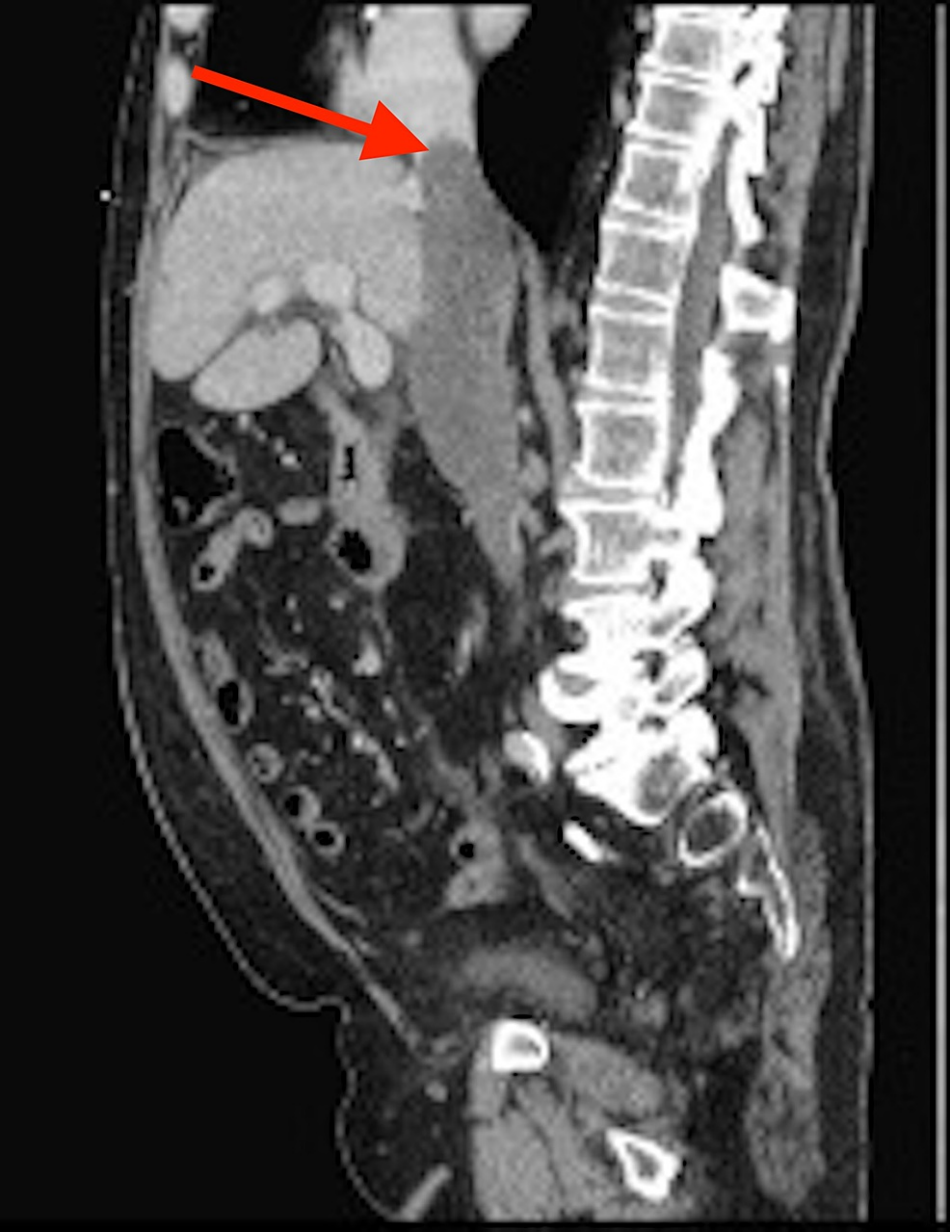
CT scan with sagittal view of inferior vena cava thrombus

A chest x-ray showed non-specific right lung base markings, but no obvious pathology. Computed tomography (CT) scan was done given smoking history and age. CT scan showed enlarged para-esophageal lymph nodes, the largest of which was 1.4x1.7 cm, a subsolid 1.0 cm nodule in the left upper lobe, and dilation of the IVC and the left renal vein concerning for thrombosis or mass. Subsequent ultrasound of the IVC showed no visualized blood flow on doppler and suggested bland versus tumor thrombus. Computed tomography angiography (CTA) showed no pulmonary embolism and further characterized the previous findings as a tumor thrombus given the associated soft tissue mass anterior to the aorta measuring 7.3x4.9 cm. Other findings on CTA included an IVC tumor thrombus 12 cm in length (Figures 1, 2) with extension into the left renal vein, along with thickening of the fundus of the stomach, reported as likely adenopathy, and multiple enlarged periaortic lymph nodes.

The oncology service was consulted and recommended biopsy with concern for lymphoma. Peri-aortic retroperitoneal lymph node biopsy was completed. Biopsy results indicated metastatic mucinous adenocarcinoma suggestive of upper gastrointestinal or pancreatic malignancy. Positron emission tomography (PET) indicated metastatic paraesophageal, retroperitoneal, and mesenteric adenopathy along with intense metabolic activity in the stomach distal to the gastroesophageal junction suspicious for primary gastric malignancy. The spleen, pancreas, and adrenal glands were unremarkable on PET scan. The patient’s case was discussed by an interdisciplinary team and recommended chemotherapy with leucovorin calcium (folinic acid), fluorouracil, and oxaliplatin and pembrolizumab. The palliative care service was consulted. After explanation of the risks and benefits of chemotherapy, the patient elected for chemotherapy in the setting of palliative care rather than a curative approach. Approximately eight weeks following the treatment plan, the patient continued to have regular follow up with both the oncology and palliative care teams. 

## Discussion

Thrombosis secondary to HCC is common, with an incidence of approximately 30% and frequently affecting the portal vein [4]. Thrombosis in gastric cancer, while rarely seen (1.2%), is similar to HCC in that the portal vein is usually affected [1]. Pancreatic cancers, while commonly pro-thrombotic, are more frequently associated with splenic, portal, or mesenteric thrombi rather than IVC [5]. Thus, IVC thrombus from gastric cancer is extremely rare. When an IVC tumor thrombus is found, the differential should remain broad and should include gastric cancers.

Presentation of a tumor thrombus is non-specific and typically by time of presentation prognosis is poor [6]. Thus, treatment is complex and frequently doesn't affect overall outcome [3]. Surgical resection remains the best option at this time, but immunotherapy, chemotherapy and radiotherapy have shown promise [3]. Thrombectomy itself has been met with mixed results and outcomes may be similar to those without thrombectomy [7]. Therefore, risk factor modification and routine cancer screening are the most appropriate methods for reducing patient presentations with a tumor thrombus.

## Conclusions

An IVC tumor thrombus is a rare finding in gastric malignancies. When seen, it is typically late in the disease course, indicating a poor prognosis. Patient presentation is variable and can range from hypotension and extremity swelling to being asymptomatic. Treatment includes treating the primary malignancy as well as the assistance of an interdisciplinary team, including palliative care, to ensure goals are understood. Since a tumor thrombus is seen late in the disease course, routine cancer screening and risk factor modification are reliable options to aid in prevention. 

## References

[1] Liang Ong SC, Batumaly SK, Jusoh SM (2018). Portal vein tumor thrombus from gastric cancer. J Ultrason.

[2] Quencer KB, Friedman T, Sheth R, Oklu R (2017). Tumor thrombus: incidence, imaging, prognosis and treatment. Cardiovasc Diagn Ther.

[3] Song Z, Wu Y, Yang J, Yang D, Fang X (2017). Progress in the treatment of advanced gastric cancer. Tumour Biol.

[4] Zanetto A, Campello E, Spiezia L, Burra P, Simioni P, Russo FP (2018). Cancer-associated thrombosis in cirrhotic patients with hepatocellular carcinoma. Cancers (Basel).

[5] Hicks AM, DeRosa A, Raj M (2018). Visceral thromboses in pancreas adenocarcinoma: systematic review. Clin Colorectal Cancer.

[6] Ozben B, Papila N, Tanrikulu MA, Bayalan F, Fak AS, Oktay A (2007). Inferior vena caval tumor thrombus extending into the right atrium in a patient with pancreatic cancer. J Thromb Thrombolysis.

[7] Tanaka A, Takeda R, Mukaihara S (2002). Tumor thrombi in the portal vein system originating from gastrointestinal tract cancer. J Gastroenterol.

